# Role of MRI in the Diagnosis of Ductal Carcinoma In Situ: A Retrospective Study

**DOI:** 10.3390/jcm14082842

**Published:** 2025-04-20

**Authors:** Cristina García Ruiz, Laila Zitan Saidi, Lucía Zambrana Aguilar, Maricela Moreira Cabrera, Carolina Carvia Ponsaille, Rosa Vázquez Sousa, Carmen Martínez Porras, Antonio Fernando Murillo-Cancho

**Affiliations:** 1 Diagnostic Radiology Attending, Department of Radiology, Torrecárdenas University, 04009 Almería, Spain; 2 Diagnostic Radiology Attending, Department of Radiology, Poniente University Hospital, 04700 Almería, Spain; ulailazitan@usal.es; 3 Diagnostic Radiology Attending, Department of Radiology, Vall d’Hebron University Hospital, 08035 Barcelona, Spain; 4 Department of Nursing, Physiotherapy and Medicine, Faculty of Health Sciences, University of Almería, 04120 Almería, Spain; amc730@ual.es

**Keywords:** DCIS, pure ductal carcinoma in situ, breast imaging

## Abstract

**Background:** The use of dynamic magnetic resonance imaging (MRI) for the evaluation, detection, and characterization of ductal carcinoma in situ (DCIS) has been increasing; however, its application in this context remains controversial and uncertain. **Materials:** A retrospective study including women with pure DCIS, confirmed between January 2012 and December 2022 using ultrasound-guided core-needle biopsy (CNB) or stereotaxy-guided vacuum-assisted biopsy (VAB), was conducted. Mammography, ultrasound (US), and MRI of DCIS lesions were evaluated according to histological grade. The size of the DCIS, as assessed by mammography, US, MRI, and final surgical histopathology, was compared using Lin’s concordance correlation and Bland–Altman plots. **Results**: A total of 144 women (mean age 55.5 ± 10.3 years) with histopathological diagnoses of pure DCIS and no evidence of infiltration in the percutaneous biopsy were included in the study. Microcalcifications were the most prevalent feature observed in mammography (82.63%). Round/punctate morphology was more common in low-grade lesions, while fine pleomorphic morphology was more frequent in medium- and high-grade lesions. Lesions manifesting as microcalcifications only on mammography were significantly associated with intermediate and high-nuclear grade DCIS (*p* = 0.005). The most common MRI manifestation of DCIS was non-mass enhancement (86.11%). A total of 141 lesions showed enhancement with MRI (sensibility 97.92%). There were no significant differences (*p* = 0.29) between negative and positive enhancement with MRI and the histological grade of the lesions. There were no significant differences (*p* = 0.49) between the type of enhancement curve with MRI and the histological grade. Preoperative MRI detected additional malignancies (multifocal, multicentric, or bilateral) in 35 patients (24.31%). **Conclusions:** DCIS demonstrated enhancement with MRI regardless of histological grade but overestimated the size of the lesions in low-nuclear-grade DCIS. Preoperative MRI identified additional malignancies (multifocal, multicentric, and bilateral lesions) in 24 patients (16.67%), which were confirmed by histopathological examination. These malignancies were either undetected or not visible with mammography and ultrasound. However, MRI also overestimated the size of the DCIS, leading to three unnecessary mastectomies in our study.

## 1. Introduction

Ductal carcinoma in situ (DCIS) is a pre-invasive breast cancer, characterized by the proliferation of malignant epithelial cells lining the terminal ductal lobular unit, without histologic evidence of invasion through the basement membrane [1,2,3].

DCIS is considered a complex lesion given the heterogeneity in its genetic, biological markers and molecular abnormalities, histopathology, imaging features, and variable potential for progression to invasive disease. This heterogeneity makes it difficult to predict the progression to invasive carcinoma and the risk of local recurrence after treatment. Therefore, the early detection and delineation of DCIS involvement are important [3,4]

The risk factors for DCIS are the same as for invasive breast cancer, including older age, Caucasian race, later age at first birth, lower number of children, increased breast density, and breast cancer family history [5].

Due to the introduction of breast cancer mammography screening, the incidence of DCIS has increased dramatically, leading to overdiagnosis and overtreatment [5,6].

The most common form of presentation of DCIS with mammography is microcalcifications [7,8]; however, it can manifest as a mass or nodule, focal asymmetry, and parenchymal distortion [5,6].

Mammographic findings may not show the actual extension of DCIS, which can extend beyond areas of calcification and lead to additional surgeries [9].

Ultrasound (US) is an essential complementary technique to mammography and magnetic resonance imaging (MRI) because it can detect an invasive component, visualize non-calcified forms of DCIS, or identify occult DCIS [10].

Breast MRI detects lesions based on patterns of angiogenesis and increased vascularity. It has been proposed as an additional imaging modality for diagnosing DCIS and defining its extent [3,11].

MRI is commonly performed in the preoperative setting for breast cancer to identify tumor extension and margins, multicentricity, and contralateral disease. Since MRI can modify the treatment and improve preoperative planning compared to other techniques, dynamic contrast-enhanced MRI has increased; however, its use in this setting remains controversial.

Histopathologic grading is the most important among the biological indicators proposed as predictors of DCIS progression. Although the histologic study establishes the nuclear grade of the lesions, the latest studies suggest that the form of presentation in MRI can be related to the histopathological characteristics of DCIS [6].

Do all DCIS lesions show enhancement with MRI, regardless of histological grade? We hypothesized that all DCIS lesions, including low-grade DCIS, would demonstrate positive enhancement with MRI, contrary to the existing literature, which suggests that only intermediate and high-grade lesions show enhancement.

In patients with DCIS, does the use of MRI in the presurgical evaluation improve the accuracy of tumor-size estimation and surgical planning compared to mammography and/or ultrasound, without increasing the risk of overdiagnosis and overtreatment?

The aims of the present study are as follows:

- To evaluate the main imaging features of DCIS;
- To compare mammography, US, and MRI findings, as well as the size of DCIS, with histopathological findings and size;
- To investigate the enhancement of DCIS with MRI regardless of histological grade and its relationship with kinetic parameters;
- To assess the usefulness of preoperative MRI in diagnosing DCIS.

## 2. Materials and Methods

### 2.1. Study Design

The study design was retrospective descriptive observational.

### 2.2. Study Sample

Female patients from the population screening program, primary care, gynecology, oncology, and general surgery consultations evaluated in the Breast Imaging Section of the Radiology Department at Torrecárdenas University Hospital between January 2012 and December 2021 were initially considered eligible for our study and were recruited from the disease database.

The inclusion criteria were as follows:

- Women with a confirmed diagnosis of pure DCIS via image-guided biopsy;
- A complete preoperative radiological evaluation including mammography, US, and MRI;
- A complete histopathological study for the surgical specimen;
- The availability of clinical and radiological follow-up.

The exclusion criteria were as follows:

- Women with concurrent invasive carcinoma confirmed by a biopsy. Patients with concurrent invasive carcinoma were excluded, as pure DCIS is defined as a pre-invasive form of breast cancer. Any evidence of infiltration or invasion disqualifies the diagnosis of pure DCIS. These restrictions were implemented to reduce bias and ensure that the study findings accurately reflect the characteristics of pure DCIS, without the influence of invasive disease;
- Incomplete preoperative imaging studies;
- Incomplete histologic data from the biopsy and/or surgical specimen;
- Women who did not undergo surgery.

See Figure 1.

### 2.3. Imaging Techniques and Procedures

- Full-field digital mammography

Full-field digital mammography was performed using the SELENIA digital breast X-ray imaging system (Hologic, Inc., Bedford, MA, USA).

The examination was performed with the patient standing, with the upper body completely exposed. All metals on the body surface were removed, and the breast tissue was fully pressed against the detector. 

Standard views (craniocaudal, mediolateral oblique) and supplementary views (magnified and focal compression) were performed, depending on the case.

- Breast US

Breast US was conducted using the Epiq Elite Diagnostic 2021 (Phillips, Amsterdam, The Netherlands) and Aplio Mx-SSA-780a (Toshiba, Tokyo, Japan) US systems and using a high-frequency linear transducer.

During the examination, the patient was in a supine position, and both sides of the breast and armpit were fully exposed.

The scan included the four quadrants, the nipple–areola area, the axillary tail, and the armpit area. 

The blood flow inside the lesion was examined with color Doppler flow imaging.

- Breast MRI

MRI was invariably performed considering the patient’s menstrual cycle (between days 7 and 14 of the cycle).

All breast MRIs were performed on a 1.5-T Siemens Magnetom Symphony Tim MR version VB19 using a 4-channel dedicated breast coil with the patient in a prone position.

Each study included the following sequences:

○ Precontrast Imaging. Before the dynamic contrast-enhanced series, the following sequences were used:
  - Axial fast spin-echo T2-weighted (TR/TE, 3800/81; matrix, 384 × 384; flip angle, 10°; slice thickness, 3 mm);
  - Fat-saturated T1-weighted;
  - Echo planar diffusion with fat saturation;
  - SPAIR with 3 values of B: 50, 400, and 800. Automatically generates the ADC (TR/TE, 5700/82; matrix, 86 × 172; flip angle, 10°; slice thickness, 4.5 mm).
○ Contrast-enhanced Imaging
○ Nonionic paramagnetic contrast material with extracellular distribution, known as Gadoteridol (ProHance of 279.3 mg/mL, 500 mM) was used intravenously. The holder of authorization is Bracco International B.V., and its registration number is 60.377;
○ At our institution, contrast enhancement was achieved by injecting a standardized dose of ProHance at a rate of 2 mL/s 0.2 mmol/kg followed by a 20–30-mL saline flush injected at the same rate; the contrast material was administered in 5 mL increments based on patient weight, ensuring that each patient received at least a single dose but not more than a double dose (median, 20 mL).

Patients with renal compromise, but with a glomerular filtration rate of 30 mL/min/1.73 m^2^ or higher, received a single dose of gadolinium contrast material. The American College of Radiology guidelines recommend the bolus administration of 0.1 mmol/kg of gadolinium-based contrast material followed by a saline flush of at least 10 mL;

○ Axial three-dimensional (3D) fat-suppressed, T1-weighted sequence VIBE spoiled gradient-echo T1- T1-weighted sequence was used for both precontrast and dynamic postcontrast imaging. The pre-and postcontrast sequence was configured as a single multiphase acquisition with a temporal resolution of 1 min 15 s per phase. The first (precontrast) phase served as a subtraction mask, followed by a pause to check the images and initiate the contrast material injection. The examination included one precontrast and five postcontrast images (TR/TE, 6.2/2.9; matrix, 225 × 320; flip angle, 10°; slice thickness, 1.1 mm);
○ Following the dynamic series, a sagittal Flash-3D fat-suppressed gradient-echo T1-weighted sequence with the body coil was performed through both breasts (TR/TE, 5.5/2.4; matrix, 256 × 288; flip angle, 10°; slice thickness, 1.4 mm).

- Percutaneous biopsy

The patients were subjected to only one biopsy technique, depending on the characteristics of each lesion. If the lesion was visible with US, an US-guided core needle biopsy (CNB) was performed; if the lesion was visible only with mammography, a stereotactic-guided vacuum-assisted biopsy (VAB) was performed. As a result, we are unable to calculate specificity or sensitivity.

○ US guided CNB: Method of choice when the lesion is best visualized with US;
○ Stereotactic VAB: Method of choice when the lesion is best visualized with mammography, such as microcalcifications (most common), architectural distortion, asymmetry, or mass without a sonographic correlate.

- Histopathological study

The Van Nuys classification system was used for the histopathological study. 

The nuclear grade of DCIS was classified as low, intermediate, or high and with or without comedo necrosis. The final nuclear grade was the higher grade between surgical histopathologic and biopsy histopathologic evaluation. The time interval between diagnostic examinations and final surgery at our hospital was a maximum of one month.

If there was no residual lesion at the surgery, the final nuclear grade was decided by the biopsy histopathology.

### 2.4. Imaging Evaluation

In this study, image measurements were obtained through a consensus-based, blinded process involving three radiologists specialized in breast imaging with 18, 14, and 12 years of experience, respectively.

The rationale behind consensus measurements was to minimize interobserver variability and ensure uniformity in lesion assessment. The blinded process was implemented as follows:

- Multiple readings: Each image was initially reviewed individually by the three radiologists, who were blinded to each other’s assessments. A subsequent group discussion was held to analyze the observed differences and reach a consensus. This approach ensures that the measurements reflect a collective interpretation based on the combined experience of the specialists;
- Consensus process moderator: During the discussions, one radiologist acted as the moderator, structuring the consensus process, and ensuring that all opinions were considered. The moderator facilitated the resolution of discrepancies and ensured that the final decisions were made fairly and objectively;
- Standardized protocol: Assessments followed a predefined protocol based on the American College of Radiology BI-RADS guidelines [12]. This protocol included the following:
  ○ Specific criteria for measuring the largest diameter of lesions;
  ○ Standardized assessment of the morphological and kinetic characteristics of images;
  ○ A systematic review of images across all modalities (mammography, US, and MRI).
- Resolution of discrepancies: In case of discrepancies between radiologists, the findings were discussed until a consensus was reached. If a unanimous decision could not be made, the final determination was based on a simple majority among the three radiologists;
- Recording of consensus: The final consensual measurements were recorded in a single database and used for statistical analysis. This ensures that the data reflect a unified standard agreed upon by the specialists.

The mammography lesions were retrospectively classified as asymmetric focal density, parenchymal distortion, nodule/mass, microcalcifications, or a combination of these features. The microcalcifications were categorized according to morphology and distribution.

MRI enhancement characteristics (morphological and kinetic) were evaluated, and the findings were compared to the relevant underlying histopathologic features.

The diameter of the lesion in millimeters was measured using mammography, US, and contrast enhanced-MRI, and compared to the final surgical histopathology.

### 2.5. Data Collection and Statistical Analysis

Electronic medical records and image databases were reviewed to collect variables, including patient age, and imaging characteristics for mammography, US, MRI, histopathologic features at biopsy, and surgical specimens.

All quantitative variables were analyzed for normality of distribution using the Shapiro–Wilk or Kolmogorov–Smirnov test, as appropriate.

The continuous data that followed normal distribution were presented as the mean standard deviation and tested using the independent sample t-test. The data that did not follow the normal distribution were presented as the medians and tested using the Mann–Whitney U test.

The categorical data were expressed as numbers with percentages and were compared by using the chi-square (χ^2^) test or Fisher exact test.

Based on the normality results, quantitative variables were compared by bivariate correlations using Pearson or Spearman.

To assess the concordance between measurements obtained by mammography, MRI, and histopathology, the Lin coefficient of concordance was calculated, along with its 95% confidence intervals (95% CI). The 95% CI was estimated using the bootstrap method, which allowed for a more precise estimation within our sample. Additionally, Bland–Altman analysis was employed to evaluate the degree of agreement between the measurement methods. The limits of agreement (LOA) were calculated, along with their 95% CI, following the methodology established by Bland and Altman (Figure 2, Figure 3 and Figure 4).

All data were anonymized using statistics software. SPSS Statistics version 26 (IBM Inc., Armonk, NY, USA) was employed for statistical analyses. A significance level of *p* < 0.05 was considered for all tests, and in the case of multiple comparisons, Bonferroni correction was applied to minimize the risk of type I errors.

- Sample size calculation

The incidence of DCIS in Spain, as reported by the Spanish Society of Medical Oncology (26.4 per 100,000 inhabitants) [13], was used to estimate the sample size. This calculation was based on the assumption that the population served by our hospital is 338,000 inhabitants.

The sample size was calculated to detect a twofold change (relative risk [RR] = 2) with a power of 80% and a significance level of 0.05 (α). The sample size calculation yielded a total of 225 participants. The availability of retrospective cases in our database determined the final sample size. After applying the exclusion criteria, the total number of patients was 144. This sample represents a group of patients with pure DCIS evaluated at our center over 10 years. While the number of patients is adequate to describe imaging features and correlate them with histology, it may be insufficient to establish more definitive associations in specific subgroups.

## 3. Results

### 3.1. Patient Characteristics

**Figure 1:**
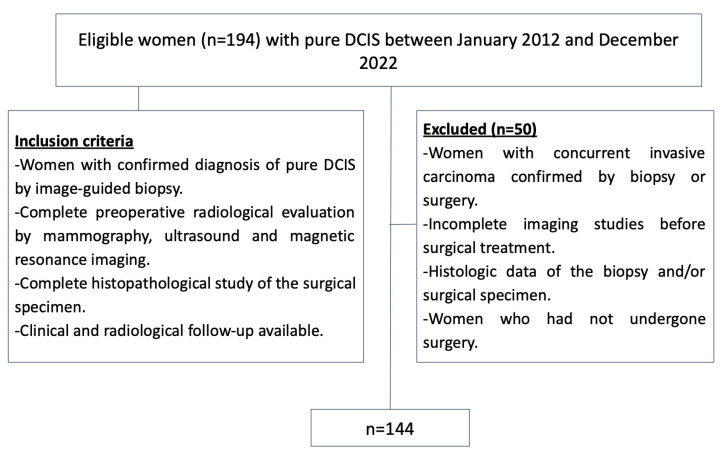
Inclusion and exclusion criteria flowchart. DCIS = ductal carcinoma in situ.

A total of 194 DCIS lesions were diagnosed through both image-guided biopsy and surgery. Among these, 50 lesions were excluded due to the following reasons: incomplete histologic data from the biopsy and/or surgical specimen (*n* = 10), lack of pre-biopsy mammograms, US, or MRI (*n* = 25), and concurrent invasive carcinoma (*n* = 15). As a result, 144 primary pure DCIS lesions in 141 patients were included in the study; three patients had bilateral DCIS lesions (Figure 1).

The median age of the 141 patients with 144 DCIS lesions was 55.5 years (interquartile range, 49.8–64 years). Of the 144 DCIS lesions, 92 lesions (63.89%) were diagnosed using ultrasound US-guided CNB while 52 lesions (36.11%) were diagnosed using stereotactic-VAB systems.

At the final histopathologic evaluation, 52 lesions (35.92%) were classified as low nuclear grade, 67 lesions (47.18%) as intermediate nuclear grade, and 25 lesions (16.90%) as high nuclear grade.

Patients’ demographic characteristics, along with baseline clinical, imaging, and pathologic data, are presented in Table 1.

### 3.2. Mammographic Features

The most frequently observed features in mammography were microcalcifications present in 82.63% of cases. Of these, 65.97% were classified as pure microcalcifications, while 16.66% exhibited microcalcifications in conjuction with other lesions, including mass/nodule, focal asymmetry, and parenchymal distortion (16.66%). The most frequent morphologic feature of microcalcifications was fine pleomorphic (54.17%), and the most frequent distribution was grouped (40.97%) and linear (21.53%).

We observed significant differences in the round and/or punctate calcification morphology (*p* = 0.02*) and fine pleomorphic morphology (*p* = 0.001*) between the patients with low-, medium-, and high-nuclear grades. Round and/or punctate morphology was more common in low-grade lesions than in medium and high grades, and fine pleomorphic morphology was more frequent in medium- and high-grade lesions.

We did not observe significant differences in the calcification distribution between the patients with low-, medium- and high-grade DCIS (Table 2), suggesting that the mammographic features of calcification are not associated with the pathologic stage of DCIS in our sample.

Lesions manifested only as microcalcifications for mammography were significantly (*p* = 0.048) associated with intermediate and high-nuclear-grade DCIS.

Lesions manifested as a mass for US without microcalcification for mammography were more frequently associated with low-nuclear-grade DCIS.

### 3.3. US Features

Ultrasound identified 99/144 lesions (68.75%) which were predominantly characterized as hypoechoic and irregular nodules (Figure 5), lesions with microcalcifications, and increased vascularization without an obvious nodule. We obtained 45 (31.25%) false negatives for US (Table 1).

### 3.4. MRI Features

The most common MRI manifestation of DCIS in our study was non-mass enhancement (86.11%) in a ductal linear (27.78%) (Figure 6), focal (27.78%), or segmental (17.36%) distribution (38.19%) with a paved internal enhancement pattern (38.19%) (Table 3).

A total of 141 lesions (97.92%) presented enhancement in MRI. Only three lesions showed no enhancement with MRI (false negatives), corresponding to the intermediate nuclear grade of DCIS (Table 3).

There were no significant differences (*p* = 0.29) between the negative or positive enhancement for MRI and the histological grade of the lesions, suggesting that the enhancement with MRI is not associated with the histopathologic grade of DCIS.

According to kinetic parameters, the most common enhancement curve was type 2 (86.23% of lesions) with no significant differences (*p* = 0.49) between the type of curve and the histological grade. The frequency of the type 2 curve was similar for the three histopathological nuclear grades (high, medium, and low). There was no type 1 curve for high nuclear-grade DCIS cases.

No significant differences were observed between the type of enhancement, internal enhancement pattern, or enhancement distribution findings for MRI and the histological grade.

A total of 35 (24.31%) patients from the eligible women showed additional disease in either breast in the MRI. We found 24 patients (16.67%) with multifocal lesions, 8 patients (5.56%) with multicentric lesions, and 3 patients (2.08%) with bilateral lesions. All additional lesions were invasive breast cancer, with no significant association with the pathologic stage of DCIS (*p* = 0.71, 0.55, 0.08 for low, intermediate, and high-nuclear-grade respectively).

### 3.5. Histological Diagnosis

Biopsy methods (core-needle and vacuum assisted) did not associate nuclear grade upgrade (*p* = 0.38) (Table 1).

A total of 31/144 (21.53%) presented necrosis foci with no significant association with the pathologic stage of DCIS (*p* = 0.15).

A total of 38 (26.39%) presented premalignant lesions with significant association (*p* = 0.0035), and 24 (46.15%) of them presented in low-nuclear-grade DCIS.

The most frequent premalignant lesion was papillomatosis/papilloma with a total of 25 (17.36%) cases. Papillomatosis/papilloma was more frequently associated with low-nuclear-grade DCIS than intermediate-nuclear-grade or high-nuclear-grade DCIS (*p* = 0.007). See Table 4.

### 3.6. Radiologic–Histopathologic Correlation

Tumor sizes ranged from 1 to 120 mm, with a median size (interquartile range) for mammography, MRI, and histopathologic evaluation of 18.0 [12.0; 40.0], 24.0 [14.0; 46.2], and 17.0 [9.5; 30.0], respectively (Table 1).

The median size for mammography and histopathologic evaluation decreased with a low nuclear grade (*p* = 0.05; *p* = 0.04) (Table 1).

Ultrasonography was not included in this comparison, as it is not routinely used to estimate tumor size in DCIS lesions. In the subgroup of low-nuclear-grade DCIS lesions, the median tumor size was 12.0 mm [7.5–23.5] for histopathologic evaluation, 15.0 mm [11.0–26.5] for mammography, and 21.5 mm [14.0–45.0] for MRI (Table 5). This pattern suggests a trend toward MRI overestimation relative to both histology and mammography. A Wilcoxon signed-rank test comparing paired MRI and mammography measurements in this subgroup revealed a statistically significant difference (Z = 0.0, *p* < 0.001), confirming that MRI tends to overestimate lesion size compared to mammography only in low-nuclear-grade DCIS.

### 3.7. Lin’s Coefficient of Concordance and Its 95% Confidence Intervals

The agreement between measurements obtained from mammography, MRI, and histopathology was evaluated using the Lin concordance correlation coefficient. The values and confidence intervals are presented in the Table 6:

### 3.8. Limits of Agreement with 95% CI (Bland–Altman)

**Figure 2:**
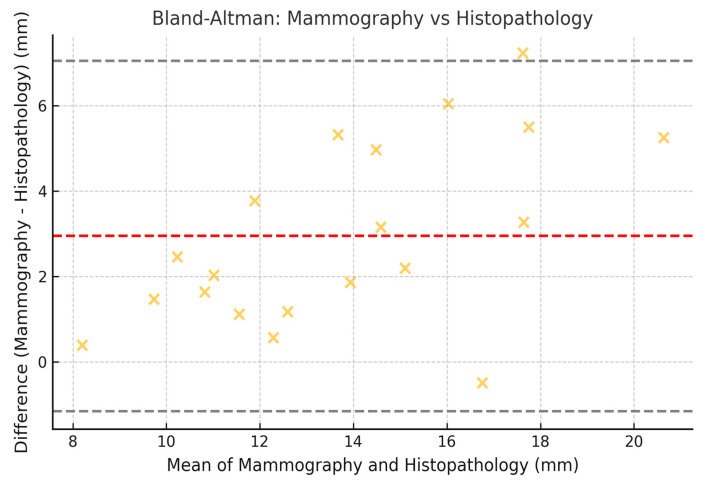
Bland–Altman plot comparing tumor size measured by mammography and histopathology. Illustrative data representing low-nuclear-grade DCIS lesions only.

**Figure 3:**
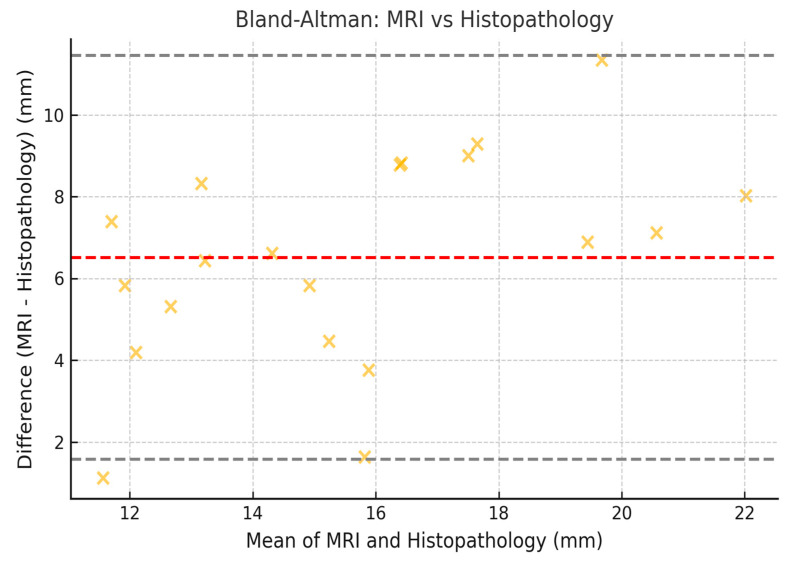
Bland–Altman plot comparing tumor size measured by MRI and histopathology. Illustrative data representing low-nuclear-grade DCIS lesions only.

**Figure 4:**
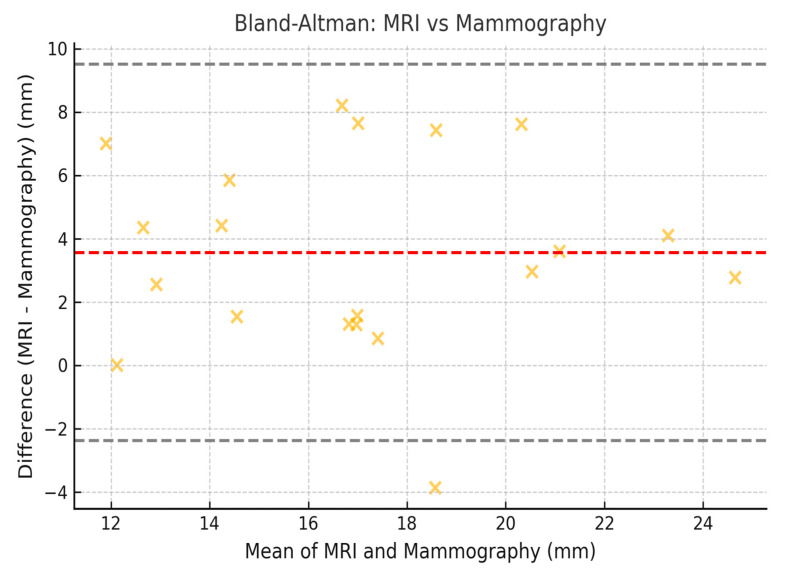
Bland–Altman correlation between the size for mammography and the size for MRI.

The Bland–Altman plots illustrate the limits of agreement between the different imaging techniques (Figure 2, Figure 3 and Figure 4). The calculated values for the limits of agreement (LOA), along with their 95% confidence intervals, are provided in the Table 7.

**Figure 5:**
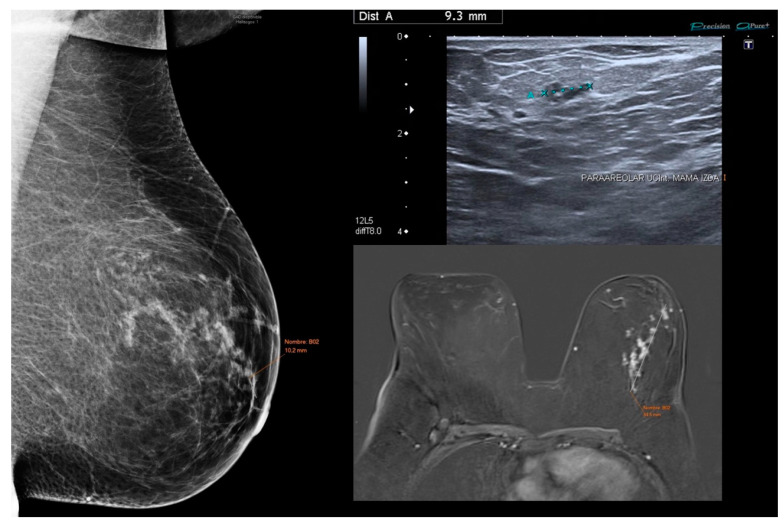
Low-grade DCIS case demonstrating variation in tumor size estimation across imaging modalities. Mammography, ultrasound, and MRI show differing lesion dimensions (10.2 mm, 9.3 mm, and 84.5 mm, respectively).

These results indicate that the highest agreement was observed between mammography and histopathology (Lin = 0.70), while the lowest agreement was found between mammography and MRI (Lin = 0.62). Regarding the limits of agreement, MRI exhibited the greatest variability in estimating tumor size compared to histopathology, with limits ranging from −10.2 mm to 14.2 mm (Figure 5).

From a clinical perspective, our findings reinforce the importance of a personalized approach in selecting presurgical studies, considering the risks associated with overestimation and the potential for unnecessary surgeries. Further research into complementary tools, such as biomarkers, is crucial for enhancing diagnostic accuracy and reducing uncertainty in the characterization of DCIS.

## 4. Discussion

We evaluated mammography, US, and MRI findings of surgically confirmed DCIS.

### 4.1. Mammography: Standard Technique with High Specificity

Mammography remains the reference modality for detecting DCIS, especially in cases with microcalcifications. It demonstrated better concordance with histopathology in estimating tumor size compared to MRI (Lin concordance coefficient = 0.70, 95% CI: 0.62–0.75). However, the agreement between mammography and MRI was lower (Lin = 0.62, 95% CI: 0.54–0.70), suggesting discrepancies in the assessment of tumor size between the two techniques.

Main Limitation: Mammography tends to underestimate the true extent of the disease, as DCIS can extend beyond calcified areas, increasing the risk of positive surgical margins and the need for reinterventions.

The most prevalent feature with mammography was microcalcifications (82.63%). Round and/or punctate morphologies were more common in low-grade lesions, while fine pleomorphic morphology was significantly more frequent in intermediate and high-grade lesions. Mammographic features, such as linear distribution and coarse heterogeneity, correlate with high-grade DCIS. However, in our study, few patients exhibited a linear distribution of calcifications.

Lesions manifesting only as microcalcifications were significantly associated with intermediate- and high-grade DCIS.

### 4.2. US: Complementary Method with Low Sensitivity in Pure DCIS

The US is useful for characterizing suspicious nodules and evaluating lesions in patients with dense breasts. However, its detection rate in pure DCIS without an invasive component is limited compared to mammography and MRI.

In our study, the US did not significantly contribute to the assessment of tumor extension (sensitivity 68.75%), consistent with previous studies highlighting its low sensitivity in non-palpable lesions without structural alterations. Lesions appearing as a mass for US without microcalcifications for mammography were more frequently associated with low-nuclear-grade DCIS. Previous studies have shown a similar association.

### 4.3. Radiologic-Histopathologic Findings

A total of 38 DCIS lesions presented premalignant features, with a significant association with low-nuclear-grade DCIS. The most frequent premalignant lesion was papillomatosis/papilloma, which was more frequently associated with low-nuclear-grade DCIS.

A comparative analysis of lesion size across imaging modalities revealed that MRI tends to overestimate tumor size relative to the low-nuclear-grade DCIS final histopathology (median: 24.0 mm vs. 17.0 mm, Table 1), a finding consistent with previous studies [10]. In contrast, mammography demonstrated a median size closer to the histopathology (18.0 mm). This observation aligns with the Bland–Altman analysis, which showed wider limits of agreement between MRI and histology compared to mammography and histology.

Although the difference in tumor size between MRI and mammography was statistically significant, the average discrepancy was only a few millimeters, suggesting that the clinical relevance of this difference should be interpreted with caution. The modest discrepancy between MRI-derived lesion size and excised tumor size may, in part, be attributable to MRI overestimation, particularly in patients with significant periductal and stromal fibrosis [14,15,16,17]. Lesions exhibiting segmental non-mass enhancement or periductal fibrosis may also contribute to size overestimation on MRI, although further investigation is warranted.

From a clinical perspective, our findings emphasize the importance of a personalized approach when selecting presurgical imaging studies, taking into account the risks of overestimation and the potential for unnecessary surgical interventions. Future research exploring complementary diagnostic tools, such as biomarkers, is essential for enhancing diagnostic accuracy and reducing uncertainty in the characterization of DCIS.

### 4.4. MRI Findings

**Figure 6:**
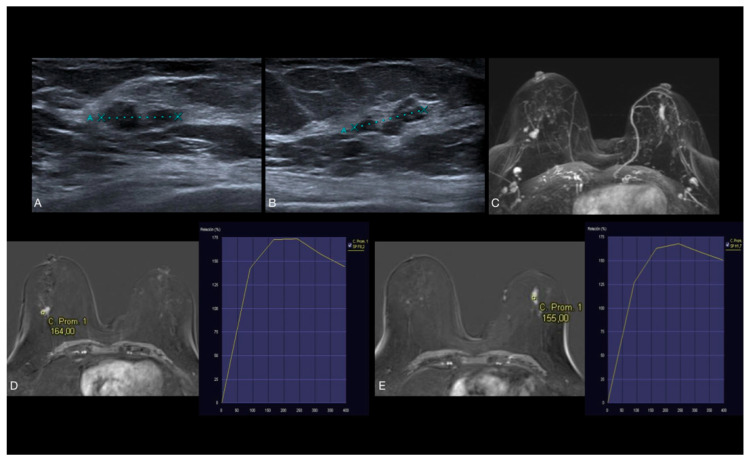
(**A**,**B**) US shows hypoechoic lobulated lesion in both breasts. (**C**–**E**) Axial early postcontrast T1-weighted MR images show well-defined mass-like enhancement in the right breast (14 mm) and ductal non-mass-like enhancement in the left breast (20 mm), both with type 2 dynamic curve. US-guided biopsy was performed. The histopathologic diagnosis of both lesions revealed low-grade DCIS without necrosis.

According to the literature, enhancement with MRI is typically observed in intermediate- and high-grade DCIS lesions [10]. However, in our study, all DCIS lesions, including those classified as low-grade, demonstrated positive enhancement with MRI, with the most frequent pattern being a type 2 curve (Figure 6). Notably, 86.23% of lesions exhibited this curve, with no significant differences (*p* = 0.49) between the type of curve and histological grade.

These findings align with those of previous studies [18,19]. This suggests that MRI enhancement may not be closely associated with the histopathologic grade of DCIS.

**Figure 7:**
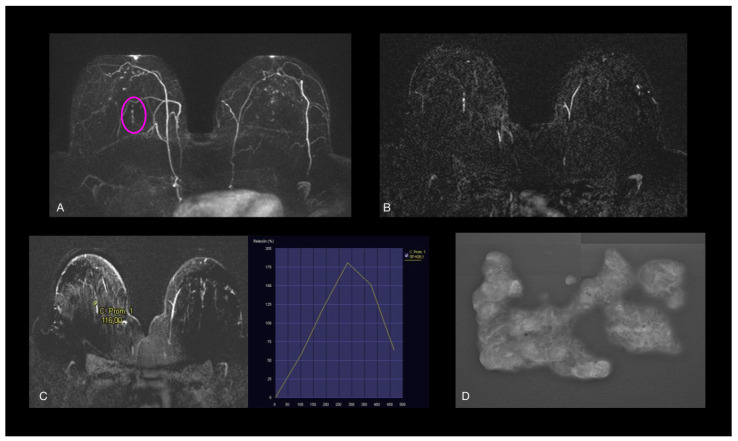
(**A**,**B**) Axial early postcontrast T1-weighted MR images show linear enhancement (pink oval), with (**C**) type 3 dynamic curve, which measured 20 mm in long-axis diameter. No US features. (**D**) Stereotactic biopsy revealed low-grade nuclear DCIS with necrosis.

The most common MRI manifestation of DCIS in our study was non-mass enhancement (86.11%), which was distributed in ductal linear (27.78%) (Figure 7), focal (27.78%), or segmental (17.36%) patterns, with a paved internal enhancement pattern in 38.19% of cases. These findings align with those reported in previous studies [20].

There was a significantly higher internal enhancement pattern with clustered rings in high-nuclear-grade lesions (*p* = 0.03), which is consistent with other studies where micro-infiltration foci were significantly associated with clustered ring enhancement [14].

The sensitivity of MRI for detecting DCIS has increased due to the use of higher-resolution sequences, which can demonstrate enhancement in both calcified and non-calcified DCIS [20].

In our study, MRI demonstrated a higher sensitivity for detecting DCIS compared to mammography and US, which aligns with previous studies reporting higher detection rates, especially in non-calcified lesions and in patients with high breast density.

The sensitivity of MRI in our study was 97.92%, higher than the 88% reported in the literature [20], as all DCIS lesions except for three showed IV contrast uptake with MRI.

### 4.5. Limitations of MRI

Despite its high sensitivity, MRI tends to overestimate tumor size in low-nuclear-grade DCIS, which may lead to unnecessary aggressive treatments like mastectomies instead of more conservative approaches, such as lumpectomy [1,15,16]. In our study, three patients underwent unnecessary mastectomies due to MRI overestimation. Two out of the three unnecessary mastectomies occurred in patients with low-nuclear-grade DCIS, supporting the concern that MRI overestimation in this subgroup may lead to overtreatment.

Bland–Altman plots confirmed discrepancies, showing that MRI tends to overestimate tumor size in low-nuclear-grade DCIS compared to histopathology, with wider limits of agreement (LOA: −10.2 mm to 14.2 mm). This discrepancy may be due to nonspecific peritumoral enhancement, likely due to the presence of periductal fibrosis or inflammatory changes [20]. The Bland–Altman results also reflect significant variability in estimating tumor size, underscoring the need for multimodal strategies in surgical planning.

In clinical practice, averaging tumor size measurements obtained from mammography and MRI may help mitigate the overestimation risk associated with MRI alone.

### 4.6. Preoperative MRI and Detection of Additional Malignancies

Despite the limitations of MRI, it is crucial for detecting additional malignancies. In our study, preoperative MRI detected multifocal, multicentric, and bilateral lesions in 24 patients (16.67%) that were missed or not visible on mammography and US. These findings included 24 patients with multifocal lesions (16.67%), 8 patients with multicentric lesions (5.56%), and 3 patients with bilateral lesions (2.08%). No significant association was found between these additional malignancies and the pathologic stage of DCIS (*p* = 0.71; *p* = 0.55; *p* = 0.08).

Previous studies, such as Tseng et al. [17], have also reported that preoperative MRI improves accuracy in assessing DCIS for a third of patients who undergo preoperative mammography and US. Early detection of DCIS is crucial because a significant proportion of cases may progress to invasive carcinoma [16]. An accurate assessment of DCIS extent is essential for successful treatment, particularly in breast conservation therapy [21,22,23].

### 4.7. The Role of MRI in Surgical Planning

Preoperative MRI can influence surgical planning by identifying additional biopsies, more extensive lumpectomies, or conversion to mastectomy [24]. According to Davis et al., surgical management was altered in 9.1% of cases [25]. Additionally, a previous meta-analysis demonstrated that 15.7% of women had a change in their surgical treatment based on preoperative MRI findings [1,26].

Some studies recommend preoperative MRI to better delineate disease margins before surgery, potentially reducing the frequency of positive margins, although this remains unproven [16,20,24].

The role of routine preoperative MRI is still under discussion due to possible overdiagnosis, false-positive findings, and unnecessary resections in patients with newly diagnosed breast cancer [27,28,29]

### 4.8. When Should MRI Be Indicated in DCIS?

Our findings emphasize the need for a personalized approach in selecting presurgical imaging studies, considering the benefits MRI offers in detecting additional tumoral disease not visible with mammography or ultrasound and the risks associated with tumor-size overestimation leading to unnecessary mastectomy.

Given the balance of benefits and limitations, we recommend using MRI selectively instead of for routine use, prioritizing it in cases where other imaging techniques are insufficient, as Lee et al. [30] have previously described:

- Suspected extensive or multifocal DCIS, where mammography’s assessment of extension is limited;
- Dense breasts, where mammography has lower sensitivity;
- Discordance between mammography and US, causing diagnostic uncertainty;
- Need to rule out an invasive component, especially in extensive lesions with non-homogeneous enhancement.

Integrating imaging biomarkers and artificial intelligence tools could enhance MRI specificity, reducing the impact of overdiagnosis and optimizing its clinical usefulness [31,32].

## 5. Conclusions

DCIS demonstrated enhancement with MRI, irrespective of histological grade, including in low-grade cases, with a high degree of sensitivity.

The role of MRI in the evaluation of DCIS remains a subject of debate, due to its high sensitivity but limited specificity. MRI can provide a detailed assessment of tumor extent and can depict additional malignancies (16.67% of patients in our study). The use of preoperative MRI in DCIS lesions should be considered on a selective basis, weighing its diagnostic benefits against the potential risks of overtreatment.

### Limitations

Our study has several limitations. First, it was a retrospective study conducted at a single institution, which introduces the potential for selection bias. Second, there was interobserver variability in the imaging evaluation. Third, the relatively small sample size may limit the generalizability of our findings, particularly in comparisons between histologic grades and in assessing the accuracy of different imaging modalities.

Larger, prospective studies are needed to confirm these results and more accurately evaluate the utility of MRI in the detection and characterization of DCIS.

## Figures and Tables

**Table 1:** Patients’ demographic, clinical, imaging, and pathologic characteristics. ^1^ One-way analysis of means. ^2^ Fisher’s Exact Test. ^3^ Pearson’s Chi-squared test. ^4^ Kruskal–Wallis rank sum test. H-B: Holm–Bonferroni correction. * Statistically significant.

Variable	Low-Nuclear Grade	Intermediate-Nuclear Grade	High-Nuclear Grade	Total	*p* Value	*p* Value (H-B)
No. of lesions	52 (35.92%)	67 (47.18%)	25 (16.90%)	144	NA	NA
Median age at diagnosis (IR) Y	55.5 (49.8–64)	55 (48–62)	50 (48–58)	55 (48–62)	0.23 ^1^	1
Mean age at diagnosis (std) Y	56.5 (10.1)	54.8 (10.4)	51.8 (9.1)	55.1 (10.3)		
Patients’ origin						
Population screening program	25 (48.08%)	34 (50.75%)	13 (52.00%)	72 (50.00%)		
Gynecology consultation	9 (17.31%)	8 (11.94%)	5 (20.00%)	22 (15.28%)		
General surgery consultation	4 (7.69%)	2 (2.99%)	0 (0%)	6 (4.17%)	0.77 ^2^	1
Oncology consultation	6 (11.54%)	8 (11.94%)	5 (20.00%)	19 (13.19%)		
Primary care consultation	6 (11.54%)	12 (17.91%)	2 (8.00%)	20 (13.89%)		
Other	2 (3.85%)	3 (4.48%)	0 (0%)	5 (3.47%)		
Biopsy method						
Core-needle biopsy	35 (67.31%)	39 (58.21%)	18 (72.00%)	92 (63.89%)	0.38 ^3^	1
Vacuum-assisted biopsy	17 (32.69%)	28 (41.79%)	7 (28.00%)	52 (36.11%)		
Lesion size median [IR] (mm)						
Mammography	15.0 [11.0; 26.5]	21.0 [12.0; 45.0]	23.0 [15.0; 53.0]	18.0 [12.0; 40.0]	0.05 *^,4^	0.50
MRI	21.5 [14.0; 45.0]	21.0 [13.5; 47.5]	26.0 [16.0; 50.0]	24.0 [14.0; 46.2]	0.66 ^4^	1
Histopathologic evaluation	12.0 [7.5; 23.5]	18.5 [10.0; 30.0]	24.5 [10.0; 40.5]	17.0 [9.5; 30.0]	0.04 *^,4^	0.44
Imaging finding						
Microcalcifications only (mammography)	26 (50.00%)	53 (79.10%)	16 (64.00%)	95 (65.97%)	0.004 *^,3^	0.048
Microcalcifications+ Asymmetric Focal Density	8 (15.38%)	7 (10.45%)	5 (20.00%)	20 (13.89%)	0.43 ^2^	1
Microcalcifications+ Parenchymal distortion	1 (1.92%)	1 (1.49%)	1 (4.00%)	3 (2.08%)	0.58 ^2^	1
Microcalcifications+ Nodule/mass	1 (1.92%)	0 (0%)	0 (0%)	1 (0.69%)	0.53 ^2^	1
Nodule/mass only (US)	6 (11.54%)	2 (2.99%)	0 (0%)	8 (5.56%)	0.09 ^2^	0.094
Parenchymal distortion	3 (5.77%)	1 (1.49%)	1 (4.00%)	5 (3.47%)	0.40 ^2^	1
Asymmetric Focal Density	4 (7.69%)	2 (2.99%)	1 (4.00%)	7 (4.86%)	0.60 ^2^	1
US						
Positive findings	37 (71.15%)	44 (65.67%)	18 (72.00%)	99 (68.75%)	0.76 ^3^	0.82
Negative findings	15 (28.85%)	23 (34.33%)	7 (28.00%)	45 (31.25%)		
MRI: Presence of enhancement						
No	0 (0%)	3 (100.00%)	0 (0%)	3 (2.08%)	0.29 ^2^	0.35
Yes	52 (36.88%)	64 (45.39%)	25 (17.73%)	141 (97.92%)		

**Table 2:** Morphology and distribution of the microcalcifications according to pathological staging. ^1^ Fisher’s Exact Test for Count Data. ^2^ Pearson’s Chi-squared test. H-B: Holm–Bonferroni correction. * Statistically significant.

Indicators	Pathological Staging: Low	Pathological Staging: Medium	Pathological Staging: High	Total	*p* Value	*p* Value (H-B)
Morphologic feature of microcalcifications						
Round/Punctiform	7 (13.46%)	1 (1.49%)	1 (4%)	9 (6.25%)	0.02 *^,1^	0.04
Amorphous	8 (15.28%)	7 (10.45%)	4 (16%)	19 (13.19%)	0.68 ^1^	1
Coarse heterogeneous	5 (9.62%)	4 (5.97%)	0 (0%)	9 (6.25%)	0.31 ^1^	1
Fine pleomorphic	17 (32.69%)	45 (67.16%)	16 (64%)	78 (54.17%)	0.001 *^,2^	0.01
Linear branched or fine linear	1 (1.92%)	5 (7.46%)	2 (8.00%)	8 (5.56%)	0.39 ^1^	1
Microcalcifications distribution						
Diffuse	1 (1.92%)	0 (0%)	0 (0%)	1 (0.69%)	0.53 ^1^	1
Regional	1 (1.92%)	5 (7.46%)	3 (12%)	9 (6.25%)	0.19 ^1^	1
Grouped	22 (42.31%)	27 (40.30%)	10 (40%)	59 (40.97%)	0.97 ^2^	1
Linear	7 (13.46%)	18 (26.87%)	6 (24%)	31 (21.53%)	0.22 ^2^	1
Segmental	7 (13.46%)	12 (17.91%)	4 (16%)	23 (15.97%)	0.83 ^1^	1

**Table 3:** Morphological and kinetic characteristics of enhancement of DCIS lesions for MRI according to the histological grade.

Variable	Low-Nuclear Grade	Intermediate-Nuclear Grade	High-Nuclear Grade	Total	*p* Value	*p* Value (H-B)	Lower 95% CI	Upper 95% CI
MRI Enhancement								
No	0 (0%)	3 (100.00%)	0 (0%)	3 (2.08%)	0.29 ^1^	1	0.14	0.44
Yes	52 (36.88%)	64 (45.39%)	25 (17.73%)	141 (97.92%)				
Curve type								
1	3 (5.88%)	3 (4.69%)	0 (0%)	6 (4.35%)	0.49 ^1^	1		
2	43 (84.31%)	57 (89.06%)	19 (82.61%)	119 (86.23%)			0.34	0.64
3	5 (9.80%)	4 (6.25%)	4 (17.39%)	13 (9.42%)				
Enhancement type								
Nodular/mass	8 (15.38%)	4 (5.97%)	2 (8%)	14 (9.72%)	0.25 ^1^	1	0.1	0.35
Non mass	41 (78.85%)	60 (89.55%)	23 (92.00%)	124 (86.11%)	0.17 ^1^	1	0.02	0.32
RFocus	1 (1.96%)	0 (0%)	0 (0%)	1 (0.69%)	0.53 ^1^	1	0.38	0.68
Internal enhancement pattern								
Paved	17 (32.69%)	31 (46.27%)	7 (28.00%)	55 (38.19%)	0.16 ^2^	1	0.01	0.31
Heterogeneous	19 (36.54%)	17 (25.37%)	7 (28.00%)	43 (29.86%)	0.41 ^2^	1	0.26	0.56
Homogeneous	15 (29.41%)	15 (28.85%)	16 (23.88%)	6 (24.00%)	0.81 ^2^	1	0.66	0.96
Clustered Ring	0 (0%)	0 (0%)	2 (8%)	2 (1.39%)	0.03 *^,1^	0.33	−0.12	0.18
Enhancement distribution								
Focal	16 (30.77%)	16 (23.88%)	8 (32.00%)	40 (27.78%)	0.62 ^2^	1.0	0.47	0.77
Linear	10 (19.23%)	25 (37.31%)	5 (20.00%)	40 (27.78%)	0.06 *^,2^	0.60	0.45	0.75
Segmental	11 (21.15%)	11 (16.42%)	3 (12.00%)	25 (17.36%)	0.65 ^1^	1	0.50	0.80
Regional	3 (5.77%)	6 (8.96%)	3 (12.00%)	12 (8.33%	0.62 ^1^	1	0.47	0.77
Multi regional	2 (3.85%)	2 (2.99%)	2 (8.00%)	6 (4.17%)	0.47 ^1^	1	0.32	0.32
Diffuse	0 (0%)	0 (%)	0 (%)	0 (0%)	NA			
Multifocality	9 (17.31%)	11 (16.42%)	4 (16.00%)	24 (16.67%)	0.96 ^1^	1	0.81	1.11
Multicentricity	3 (5.77%)	3 (4.48%)	2 (8.00%)	8 (5.56%)	0.82 ^1^	1	0.67	0.67
Bilaterality	3 (5.88%)	0 (0%)	0 (0%)	3 (2.08%)	0.23 ^1^	1	0.08	0.38

^1^ Fisher’s Exact Test for Count Data; ^2^ Pearson’s Chi-squared test. H-B: Holm–Bonferroni correction. * Statistically significant.

**Table 4:** Types of premalignant lesions found according to the histological grade of DCIS.

Variable	Low-Nuclear Grade	Intermediate-Nuclear Grade	High-Nuclear Grade	Total	*p* Value	*p* Value (H-B)
Presence of premalignant lesions	24 (46.15%)	11 (16.42%)	3 (12.00%)	38 (26.39%)	0.005 *^,1^	0.035
Presence of necrosis foci	8 (25.8%)	12 (38.7%)	11 (35.5%)	31 (21.53%)	0.15 ^2^	1
Premalignant lesions						
Papillomatosis/Papilloma	19 (36.54%)	4 (5.97%)	2 (8.00%)	25 (17.36%)	0.001 *^2^	0.007
Atypical ductal hyperplasia	0 (0%)	0 (0%)	1 (4%)	1 (0.69%)	0.17 ^2^	1.0
Atypical squamous epithelium	1 (1.96%)	2 (2.99%)	0 (0%)	3 (2.08%)	1.0 ^2^	1.0
Complex sclerosing lesion	0 (0%)	1 (1.49%)	0 (0%)	1 (0.69%)	1.0 ^2^	1.0
Lobular carcinoma in situ	1 (1.92%)	0 (0%)	0 (0%)	1 (0.69%)	0.53 ^2^	1.0
>1	3 (5.77%)	4 (5.97%)	0 (0%)	7 (4.86%)	0.68 ^2^	1.0

^1^ Pearson’s Chi-squared test. ^2^ Fisher’s Exact Test for Count Data. H-B: Holm–Bonferroni correction. * Statistically significant

**Table 5:** Median tumor size in low-nuclear-grade DCIS lesions measured by mammography, MRI, and final histopathology. Only lesions classified as low nuclear grade were included in this analysis to prevent overgeneralization across different tumor grades.

Image Modality	Median (IQR), mm	Wilcoxon Test (*p* Value)
Mammography	15.0 [11.0–26.5]	
MRI	21.5 [14.0–45.0]	0.0 (*p* < 0.001)
Histopathological evaluation	12.0 [7.5–23.5]	

**Table 6:** Agreement between mammography, MRI, and histopathology measurements.

Comparison	Lin Coefficient	Lower CI 95%	Upper CI 95%
Mammography vs. Histopathology	0.70	0.62	0.75
MRI vs. Histopathology	0.69	0.61	0.74
Mammography vs. MRI	0.62	0.54	0.70

**Table 7:** Limits of agreement and confidence intervals for imaging techniques.

Comparison	Lower Limit	Upper Limit	Lower CI 95%	Upper CI 95%
Mammography vs. Histopathology	−8.3 mm	11.3 mm	−9.1 mm	12.1 mm
MRI vs. Histopathology	−10.2 mm	14.2 mm	−11.0 mm	15.0 mm
Mammography vs. MRI	−7.5 mm	9.1 mm	−8.2 mm	9.8 mm

## Data Availability

The original contributions presented in this study are included in the article. Further inquiries can be directed to the corresponding author.

## Abbreviations

DCIS	Ductal carcinoma in situ
CNB	Core-needle biopsy
MRI	Magnetic resonance imaging
US	Ultrasound
VAB	Stereotaxy-guided vacuum-assisted biopsy
